# Evaluating muscle aging during relaxed standing, squats and lunges through electrical bioimpedance

**DOI:** 10.1038/s41598-025-27187-3

**Published:** 2025-11-10

**Authors:** Samaneh Zolfaghari, Abdelakram Hafid, Saad Abdullah, Annica Kristoffersson, Mia Folke

**Affiliations:** School of Innovation, Design and Engineering, Mälardalen University, 722 20 Västerås, Sweden

**Keywords:** Electrical bioimpedance, Physical activities, Muscle aging, Muscle function, Feature analysis, Statistical analysis, Biomedical engineering, Quality of life

## Abstract

Electrical bioimpedance (EBI) is widely used for body composition analysis and shows promise for assessing muscle activation during physical activities (PAs), particularly in aging. This study investigated EBI’s sensitivity to age-related changes in muscle function by analyzing data from 40 adult participants divided into young (20–29 years), middle-aged (32–60 years), and older (62–73 years) groups. EBI signals were recorded from the Quadriceps and Extensor Digitorum Longus (EDL) muscles during three PAs: relaxed standing position, squats, and lunges. Key features were extracted to identify age-related differences. Results revealed distinct muscle-specific patterns: In the relaxed standing position, the EDL muscle exhibited a consistent, monotonic decline in the *PrePAmagnitude* feature from young to old adults, while the Quadriceps muscle displayed greater variability and a non-monotonic trend. Among the dynamic activities, squats revealed the most pronounced age-related differences, with 62.5% of the features showing statistical significance, whereas fewer differences in the features (25%) where shown during lunges. The findings suggest that EBI can detect age-related reductions in muscle activation and neuromuscular coordination, supporting its potential as a non-invasive tool for functional muscle assessment in aging.

## Introduction

Sports and exercise medicine is a specialized field focused on optimizing physical performance, preventing injuries, and supporting rehabilitation in athletes^1^. While sports medicine is traditionally associated with competitive sports, the field also encompasses the study of fundamental movement functions, such as balance and mobility, which are crucial for both athletic performance and injury prevention^2,3^. These functions tend to decline with age due to factors such as neuromuscular degeneration and loss of muscle strength, increasing the risk of injuries^2^. In this regard, to understand and improve the subtleties of balance and muscle function across different age groups, measurement techniques to assess early age-related declines in physical ability are important.

Different wearable sensors are used to evaluate age-related changes in execution of physical activity (PA), as well as balance and muscle function. Inertial measurement units have limitations in detecting early age-related declines in how to execute a PA^4^ and cannot assess muscle engagement or the muscle-status. Electromyography (EMG) measures electrical activity during muscle contractions and provides insights into neuromuscular function and supports the diagnosis of disorders and muscle fatigue^5,6^. EMG has been used to identify age-related changes in muscle activity, such as decreased amplitude, shifts in frequency content, and changes in motor unit firing patterns^7,8^, which reflect the neuromuscular remodeling that occurs with aging, including a reduction in motor unit numbers and an increase in motor unit size^8^. Electrical Bioimpedance (EBI) has emerged as a promising measurement tool for evaluating muscle activity during PAs. EBI measures the electrical properties of muscle tissue, allowing for continuous monitoring of muscle activation function and PA analysis^9–11^. Additionally, EBI provides complementary physiological insights by capturing changes in muscle composition^12^ and hydration^13^. However, variations in tissue composition and external factors can impact signal quality and interpretation of EBI signals^10,14^. Despite these challenges, EBI remains a promising tool for real-world applications in sports and rehabilitation where non-intrusive and reliable monitoring of muscle function is essential. However, no studies on age-related declines in muscle function measured with EBI have been found.

In our previous study^15^, the potential of the EBI technique for analyzing muscle activation during the PAs squats, lunges, balance walk and short jump was explored with data obtained from the Quadriceps muscle and the Extensor digitorum longus (EDL) muscle. Our findings demonstrated that individual movement cycles could be automatically extracted from the EBI signals obtained while performing squats and lunges and that each of the four PAs exhibited distinct EBI signal characteristics. It was also observed that the EBI signal characteristics varied between participants but also between each PA cycle for individual participants. The data collected showed that the EBI signals obtained at the EDL muscle and Quadriceps muscle provided evidence that they are both active while performing different ADLs such as walking, sitting, and standing. The data collected also showed that variations in the EBI signals occurred due to coordination or imbalance problems. This highlighted the potential of EBI for differentiating between various PA patterns. The aim of this study was to further explore the potential of the EBI technique’s sensitivity to changes related to age by (i) collecting EBI data from 40 participants across various age groups, (ii) investigating informative features regarding our target PAs (i.e., relaxed standing position, squats and lunges), and (iii) performing an extensive analysis of PAs based on features across age groups.

## Methods

**Fig. 1:**
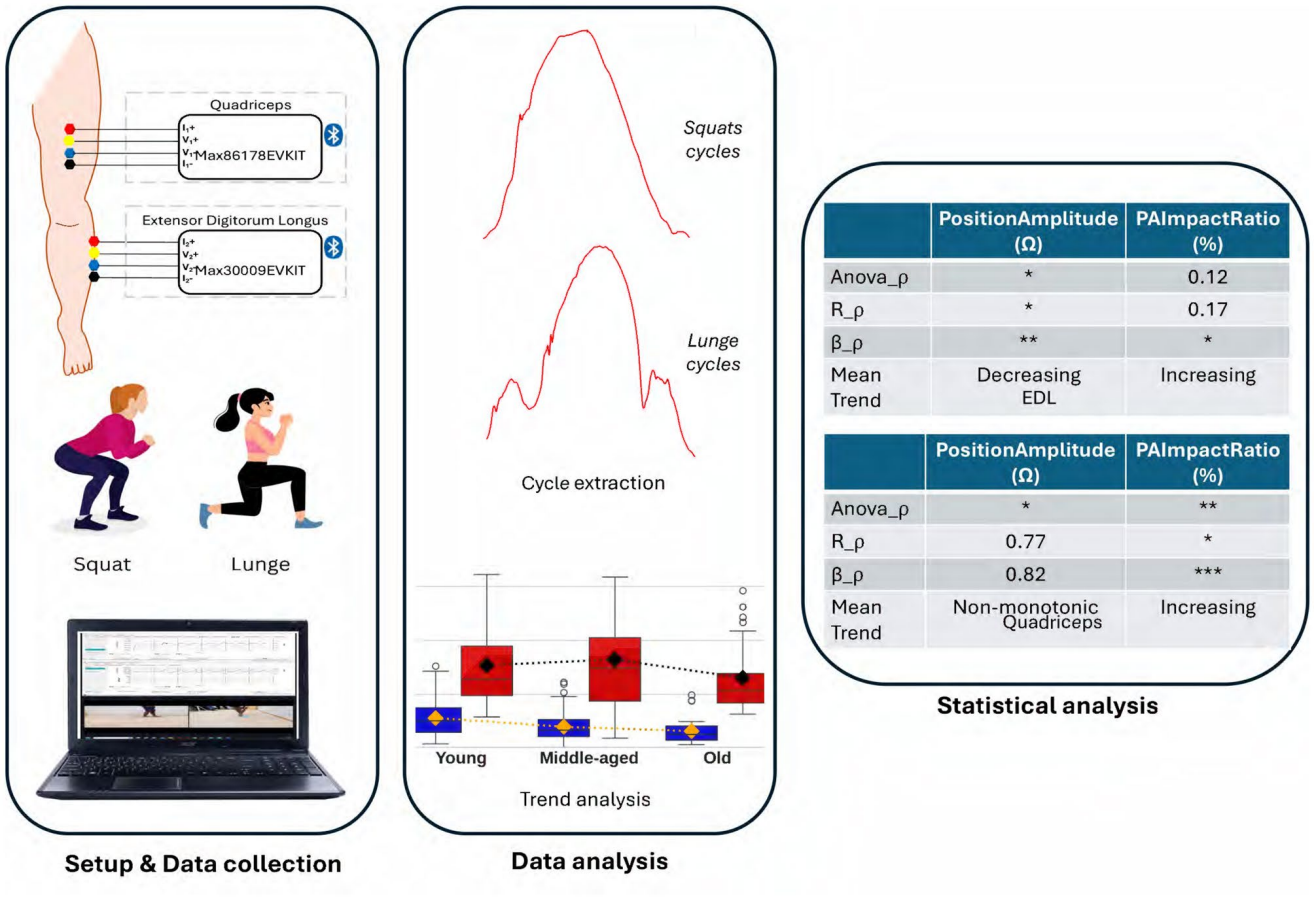
An overview of the methodology.

Figure 1 illustrates an overview of the methodology followed during data collection, and data analysis. The study was approved by the Swedish Ethical Review Authority (reference code 2022-06690-01) and adhered to the Declaration of Helsinki.

### Setup and data collection

We recruited a convenience sample of 40 participants to the study, through word of mouth and university channels. This approach resulted in demographic heterogeneity across age groups, with varying gender distributions as detailed in Table 1. The participants were divided into three age groups, namely, young, middle-aged, and old adults. All participants provided informed consent after receiving detailed information about the study.

**Table 1:** Participants’ demographics. Gender distribution is provided in % and number of participants.

Age groups	Age range	Number	Gender	
			Female	Male
Young	20–29	11	27% (3)	73% (8)
Middle-aged	32–60	19	68% (13)	32% (6)
Old	62–73	10	40% (4)	60% (6)

To establish a baseline reference impedance amplitude across age groups, impedance values were recorded in the PA relaxed standing position for 30 seconds. In addition, the participants performed squats and lunges (using the leg where the sensors were attached to step forward) while wearing eight Ag/AgCl electrodes in a tetrapolar configuration: four electrodes on the Quadriceps muscle and four electrodes on the EDL muscle. Participants received both oral and visual instructions to ensure standardized execution of the PAs as established in our previous validation work^15^:

- Squats: Participants were instructed to perform five squats as quickly as possible. Each squat involved bending the knees and descending towards a chair without sitting down, followed by a return to the standing position. The same chair has been used across all measurements. This ensured consistent squat depth and minimized variability in movement execution.
- Lunges: Participants performed three forward lunges. Each lunge required stepping forward using the leg on which the sensors were attached as front leg, and then bending the knees into a position where both knees were bent approximately 90$^\circ$, followed by a return to the starting position. The foot of the front leg was not allowed to touch the ground again until the participant had fully returned to the initial stance. This ensured that the participants executed the lunges in a consistent as possible way.

Moreover, UltraSharp Webcam (WB7022) cameras were utilized to capture the participants’ PAs. The camera angles were specifically chosen to focus on the lower part of the body. The video recordings supported the data analysis.

Detailed information on sensor locations and the execution of the PAs can be found in our previous work^15^. Participants received verbal and visual instructions on how to execute the PAs and when to start the respective PAs. There was brief resting periods between the PAs. The total time for mounting and dismounting the electrodes, connecting the cables, ensuring good signal quality and conducting the PAs was 30 minutes per participant. The EBI signals were collected with two cards, Max86178EVKIT and Max30009EVKIT (Maxim Integrated, USA). These kits are designed for high-resolution, low-power bioimpedance measurements and integrate analog front-end systems with embedded microcontroller units. Each device was powered by a rechargeable lithium-polymer battery and communicated wirelessly with a dedicated graphical user interface via Bluetooth for real-time monitoring and data acquisition. The rationale for selecting two different EBI cards, and the data acquisition protocol, is described in detail in our previous work^15^.

### Data analysis

To analyze the data, individual movement cycles were extracted for each muscle during squat and lunge activities. The cycle extraction process followed the same methodology and algorithms detailed in our previous work^15^. For consistency, three squats (cycle 2–4) and three lunges’ cycles were considered per participant.

Following cycle extraction, key magnitude-based features were computed from the recorded EBI signals to assess muscle activation patterns. These features capture critical aspects of the movement cycle, allowing for a detailed evaluation of muscle function during both dynamic PAs. The extracted features were processed offline using a custom Python program. The three features previously described in^15^, namely *BaselineToPeakAmplitude*, *PositionAmplitude*, and *NumberOfFluctuations*, were used in the analysis of the squat and lunge activities. Table 2 provides a description of these features and their physiological interpretation.

**Table 2:** Description of EBI features and their physiological interpretation.

Feature	Definition	Physiological interpretation
*BaselineToPeakAmplitude *($\Omega$)	Difference between baseline and peak impedance during a cycle.	Reflects the muscle mass.
*PositionAmplitude *($\Omega$)	Amplitude of impedance variation across the movement cycle.	Degree of muscle contraction and range of motion.
*NumberOfFluctuations*	Count of local minima and maxima within a cycle.	Neuromuscular coordination and stability.

The impedance values recorded while standing in a relaxed position for 30 seconds, defined as *PrePAmagnitude*, provides insight into muscle condition before doing the squat and lunge PAs. Additionally, the feature *PAImpactRatio* (Eq. 1) which provides insights into muscle change due to the PA, was introduced. It is defined as:

1 $$\begin{aligned} PAImpactRatio=\frac{EndlineMagnitude-BaselineMagnitude}{BaselineMagnitude}*100 \end{aligned}$$

### Statistical analysis

Global trends across age groups for each muscle were identified by analyzing the extracted features. Initially, the mean, median, and interquartile range (IQR) values of each feature for the young, middle-aged, and old adult groups were computed. Then, a series of statistical analyses to assess differences in extracted features across age groups and muscle was conducted. First, One-Way ANOVA was performed separately for each muscle to compare mean feature values among the three age groups. To evaluate the strength and direction of associations between age and feature variations, we calculated Spearman’s rank correlation coefficient (r), which assesses monotonic relationships between age groups and feature values. Finally, Ordinary Least Squares (OLS) regression was applied to quantify the effects of age group, muscle, and their interaction on each feature. Unlike ANOVA, which identifies group differences, OLS regression estimates ($\beta$) coefficients, providing insight into the magnitude and direction of these effects. Statistical significance was set at r < 0.05 for all analyses.

To account for the increased risk of Type I (false positives) errors due to multiple statistical tests, we applied the Benjamini-Hochberg procedure^16^ to control the false discovery rate (FDR) at 0.05. The adjusted $\rho$-values were calculated, and results with corrected $\rho$-values below 0.05 were considered statistically significant. This approach balances sensitivity and error control, making it suitable for our study that involve multiple statistical tests. A concise summary of the statistical methods used is provided in Table 3.

**Table 3:** Overview of the statistical methods employed.

Method	Purpose	Output
Descriptive statistics	Summarize central tendency and variability per group	Mean, Median, IQR
One-Way ANOVA	Compare mean feature values across age groups (per muscle)	F-statistic, $\rho$-value
Spearman’s rank correlation	Assess monotonic relationship between age and feature values	r, $\rho$-value
OLS regression	Quantify effects of age, muscle, and interaction on feature values	$\beta$-coefficients, $\rho$-values
Benjamini–Hochberg procedure	Control false discovery rate due to multiple testing	Adjusted $\rho$-values (FDR < 0.05

To visualize the results, box plots to illustrate feature distributions, variability and central tendencies within the data across age groups were created. Mean trend dots lines were overlaid on the box plots. Furthermore, summary tables of the statistical analysis performed were developed.

## Results

This section presents the results of EBI signal analysis based on the extracted features obtained during different PAs to examine muscle change, trends, and data distribution and statistical test across muscles and various age groups. It should be mentioned that, out of the 40 participants, the data from 39 participants was included. One middle-aged participant was excluded in the presentation of results due to abnormal feature values.

### Relaxed standing position

**Fig. 2:**
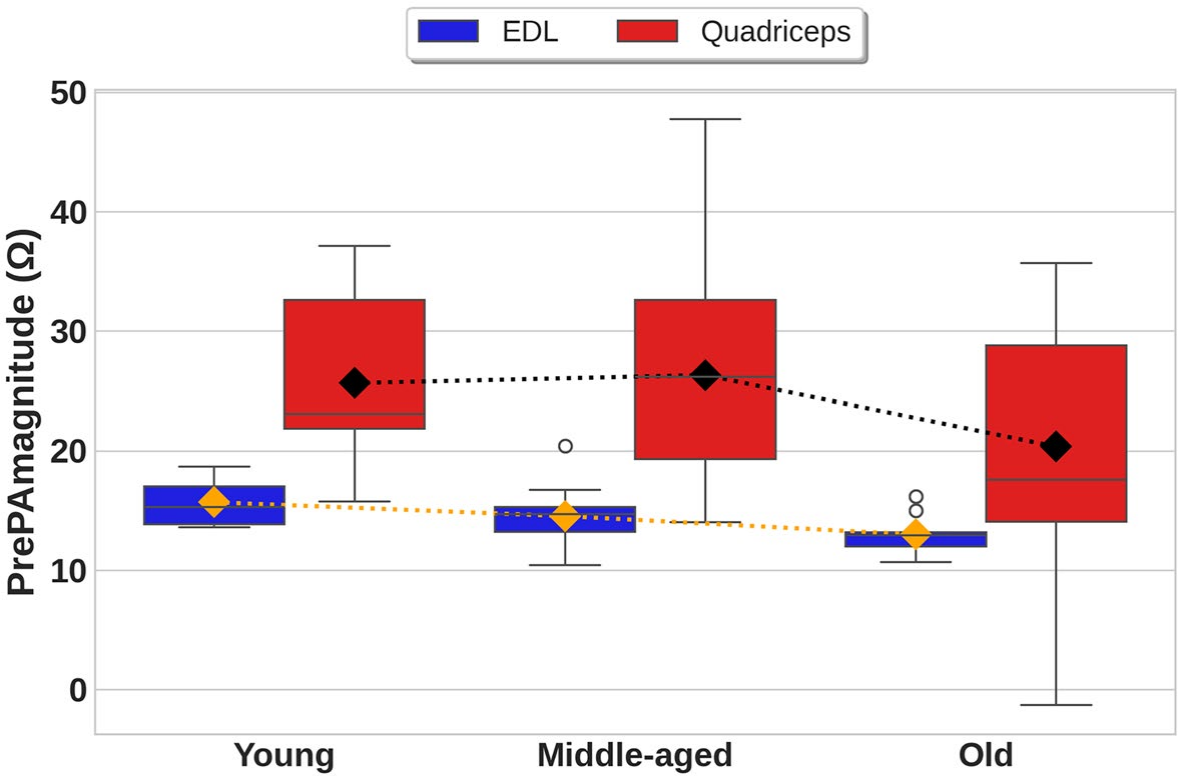
Box plots illustrating feature distribution across age groups and muscles in EBI signals during relaxed standing position. The diamonds indicate the means and the mean trend lines are indicated by dots.

Figure 2 presents box plots with overlaid mean trend lines, depicting the distribution of *PrePAmagnitude* across various age groups and muscles during relaxed standing. This visualization aids in identifying patterns and variations within the data, offering insights into the influence of age and muscle groups on EBI signals. Table 4 presents the statistical results obtained for the *PrePAmagnitude* across different age groups.

The analysis of *PrePAmagnitude*, revealed distinct age-related changes across both muscles. Specifically, the EDL muscle demonstrated a consistent, statistically significant monotonic decline in *PrePAmagnitude* when aging, dropping from 15.7 $\Omega$ in the young group to 12.99 $\Omega$ in the old group. This trend was supported by a one-way ANOVA (F = 16.53, $\rho$ $<.001$), and further corroborated by a Pearson correlation coefficient of r = $-0.53$ ($\rho$ $<.001$) and an OLS regression coefficient of $-1.35$ ($\rho$ $<.001$). Also, the relatively narrow error bars and limited spread for the EDL box plots across all age groups indicate low inter-subject variability and a robust age-related decline.

In contrast, the Quadriceps muscle exhibited a non-linear trend: mean *PrePAmagnitude* increased from 25.64 $\Omega$ in the young group to 26.31 $\Omega$ in the middle-aged group, subsequently decreasing to 20.35 $\Omega$ in the old group, This variation was statistically significant, with a one-way ANOVA (F = 4.80, $\rho$ = .01), a Pearson correlation of (r = $-0.19$ ,$\rho$ = .04), and an OLS regression coefficient of $-2.57$ ($\rho$ = .023). ). Although a correlation between age and impedance was observed for the Quadriceps muscle, the association was relatively weak, indicating a less consistent relationship compared to the EDL muscle. This pattern is further supported by the box plot analysis, which showed larger error bars and a broader data distribution for the Quadriceps muscle across all age groups, suggesting greater inter-individual variability. Notably, the Quadriceps muscle consistently exhibited higher *PrePAmagnitude* values than the EDL muscle across all age groups.

### Squats

**Fig. 3:**
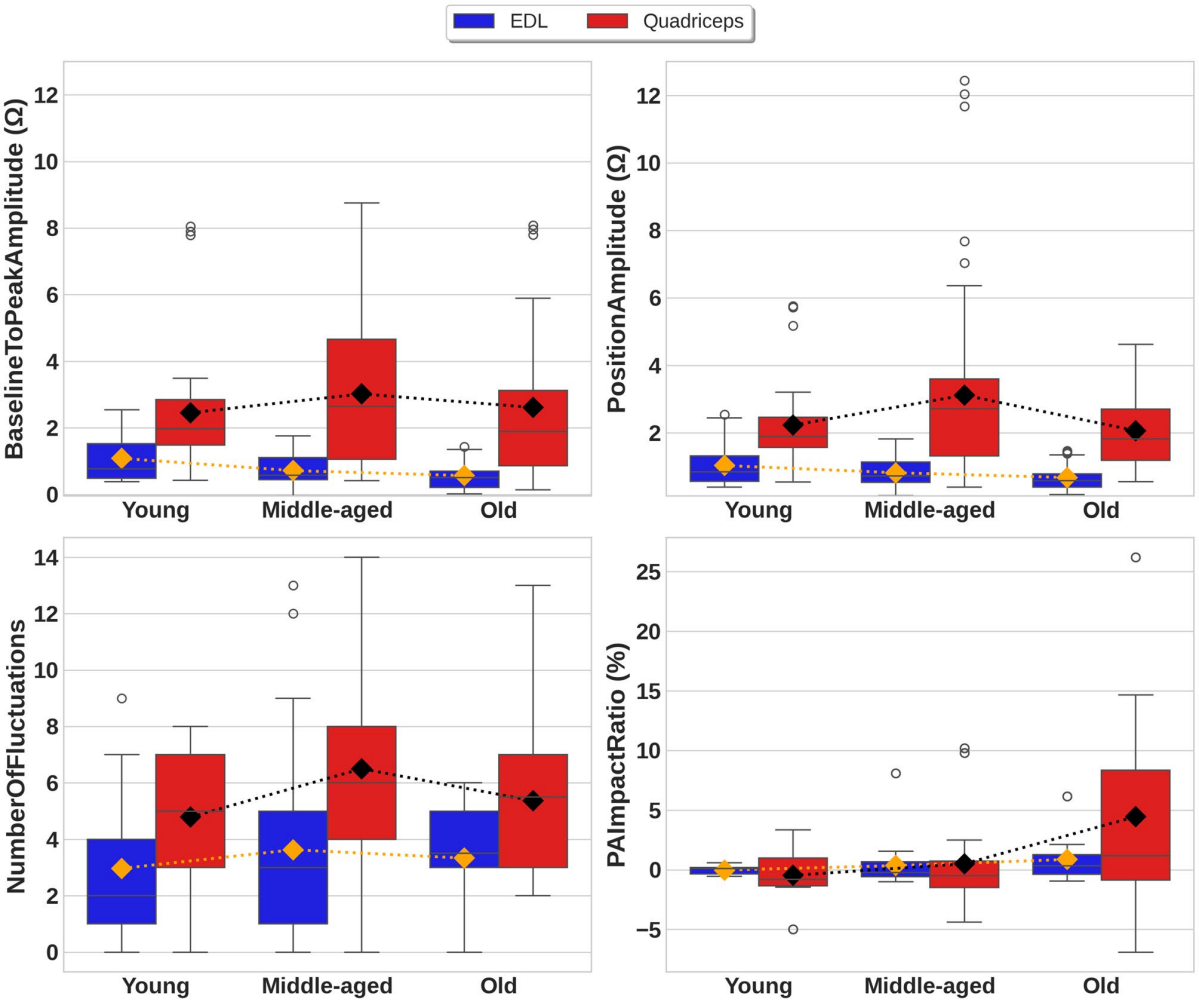
Box plots illustrating the feature distribution across age groups and muscles in the EBI signals obtained during squats. The diamonds indicate the means and the mean trend lines are indicated by dots.

Figure 3 illustrates boxplots and mean trend dot lines for the *BaselineToPeakAmplitude, PositionAmplitude, NumberOfFluctuations*, and *PAImpactRatio* across different age groups and muscles during squats.

The mean values for the two features *BaselineToPeakAmplitude* and *PositionAmplitude* show a progressive decrease from the young to old group for the EDL muscle, whereas the mean values for these two features followed a non-monotonic trend for the Quadriceps muscle, increasing between the young group and middle-aged group before decreasing between the middle-aged group and the old group. The mean values for the *NumberOfFluctuations* followed a non-monotonic trend for both muscles, increasing between the young group and the middle-aged group before decreasing between the middle-aged group and the old group.

In contrast, the mean values for *PAImpactRatio* exhibited a monotonic increase for both muscles, transitioning from negative mean values in the young group to progressively higher positive mean values in middle-aged and old group. A sharp increase can be observed between the middle-aged group and old group for the Quadriceps muscle.

Table 5 presents the statistical results obtained for the features across different age groups during squats.

The statistical analysis revealed distinct trends across features and muscles. The mean values for *BaselineToPeakAmplitude* showed a significant age-related decrease for the EDL muscle, with mean values decreasing from 1.08 $\Omega$ for the young group to 0.57 $\Omega$ for the old group. This downward trend was supported by ANOVA (F = 8.14, $\rho$ $<.001$), Spearman correlation (r=$-0.29$, $\rho$ $<.001$), and OLS regression ($\beta$ = $-0.26$, $\rho$ $<.001$). In contrast, the *BaselineToPeakAmplitude* showed a non-monotonic trend for the Quadriceps muscle, with the highest mean value for the middle-aged group (3.02 $\Omega$). No statistically significant differences were observed (ANOVA F = 0.76, $\rho$ = .47; r = $-0.02$, $\rho$ = .83; $\beta$ = 0.09, $\rho$ = .75). It is also noticeable that there is a wider spread box plot and more outliers in EBI signals from the Quadriceps muscle across all age groups, indicating greater inter-individual variability.

Similarly, the mean values for *PositionAmplitude* displayed a significant decrease for the EDL muscle. This decrease was supported by ANOVA, Spearman correlation, and OLS regression, with mean values decreasing from 1.04 $\Omega$ for the young group to 0.67 $\Omega$ for the old group. ANOVA (F = 4.32, $\rho$ = .016), Spearman correlation (r = $-0.23$, $\rho$ = .014), and OLS regression ($\beta$= $-0.18$, $\rho$ = .004) supported this downward trend. In contrast, the *PositionAmplitude* showed a non-monotonic trend for the Quadriceps muscle, with the highest mean value observed for the middle-aged group (3.12 $\Omega$). ANOVA indicated a slight difference across groups (F = 3.41, $\rho$ = .036), but Spearman correlation (r = $-0.03$, $\rho$ = .77) and OLS regression ($\beta$ = $-0.06$, $\rho$ = .82) did not support a significant trend. Again, the EBI signal from the Quadriceps muscle exhibited a larger spread and more outliers in the box plots.

The mean values for *NumberOfFluctuations* displayed a non-monotonic trend for both muscles. For the EDL muscle, the highest mean was observed in the middle-aged group (3.63). ANOVA did not detect significant differences (F = 0.74, $\rho$ = .48), nor did Spearman correlation (r = 0.10, $\rho$ = .31) or OLS regression ($\beta$ = 0.19, $\rho$ = .54). For the Quadriceps muscle, a peak was observed in the middle-aged group (6.5). ANOVA showed a significant difference across age groups (F = 4.29, $\rho$ = .016), although both Spearman correlation (r = 0.07, $\rho$ = .44) and OLS regression ($\beta$ = 0.32, $\rho$ = .37) were not statistically significant. The box plots for this feature also show that the EBI signal from the Quadriceps muscle had a consistently wider spread and more variability than the EBI signal from the EDL muscle, particularly in the middle-aged group.

For the mean values for *PAImpactRatio*, different trends were observed for the two muscles. In the EDL muscle, values showed a slight increase from $-0.03$% in the young group to 0.86% in the old group. ANOVA (F = 2.13, $\rho$ = .124) and Spearman correlation (r = 0.13, $\rho$ = .17) were not significant, but OLS regression did show a significant result ($\beta$ = 0.44, $\rho$ = .042). The Quadriceps muscle showed a strong increasing trend, with values rising from $-0.47$% in the young group to 4.45% in the old group. This trend was supported by ANOVA (F = 7.28, $\rho$ = .001), Spearman correlation (r = 0.18, $\rho$ = .053), and OLS regression ($\beta$ = 2.42, $\rho$ $<.001$).

Although some Spearman correlation coefficients reached statistical significance, such as for *BaselineToPeakAmplitude* (r = $-0.29$, $\rho$ $<.001$) and *PositionAmplitude* (r = $-0.23$, $\rho$ = .014) for the EDL muscle, and *PAImpactRatio* (r = 0.18, $\rho$ = .05) for the Quadriceps muscle, the overall r values were generally low for both muscles during squats. This indicates that while age-related trends exist, the strength of these relationships is weak to moderate.

**Table 4:** Statistical results for *PrePAmagnitude* (* < 0.05, ** < 0.01, *** < 0.001).

Feature	Muscle	Age group	Mean	Median	IQR	ANOVA_F	ANOVA_$\rho$	r	r_$\rho$	$\beta$	$\beta$_$\rho$	Mean trend
*PrePAmagnitude *($\Omega$)	EDL	Young	15.7	15.3	13.86-17.02	16.53	***	-0.53	***	-1.35	***	Decreasing
		Middle-aged	14.53	14.69	13.21-15.29							
		Old	12.99	12.9	11.98-13.17							
	Quadriceps	Young	25.64	23.07	21.85-32.61	4.8	*	-0.19	*	-2.57	*	Non-monotonic
		Middle-aged	26.31	26.17	19.31-32.61							
		Old	20.35	17.56	14.08-28.81

**Table 5:** Summary of statistical results for features from EBI signals during squats (* < 0.05, ** < 0.01, *** < 0.001).

Feature	Muscle	Age group	Mean	Median	IQR	ANOVA_F	ANOVA_$\rho$	r	r_$\rho$	$\beta$	$\beta$_$\rho$	Mean trend
*BaselineToPeakAmplitude *($\Omega$)	EDL	Young	1.08	0.77	0.48–1.52	8.14	***	-0.29	**	-0.26	***	Decreasing
		Middle-aged	0.72	0.57	0.45–1.11							
		Old	0.57	0.5	0.21–0.70							
	Quadriceps	Young	2.45	1.96	1.48–2.85	0.76	0.47	-0.02	0.83	0.09	0.75	Non-monotonic
		Middle-aged	3.02	2.65	1.06–4.66							
		Old	2.61	1.89	0.86–3.12							
*PositionAmplitude *($\Omega$)	EDL	Young	1.04	0.85	0.57–1.32	4.32	*	-0.23	*	-0.18	**	Decreasing
		Middle-aged	0.82	0.73	0.54–1.14							
		Old	0.67	0.58	0.40–0.79							
	Quadriceps	Young	2.23	1.9	1.57–2.46	3.41	*	-0.03	0.77	-0.06	0.82	Non-monotonic
		Middle-aged	3.12	2.71	1.32–3.60							
		Old	2.07	1.82	1.19–2.71							
*NumberOfFluctuations*	EDL	Young	2.97	2	1–4	0.74	0.48	0.1	0.31	0.19	0.54	Non-monotonic
		Middle-aged	3.63	3	1–5							
		Old	3.33	3.5	3–5							
	Quadriceps	Young	4.79	5	3–7	4.29	*	0.07	0.44	0.32	0.37	Non-monotonic
		Middle-aged	6.5	6	4–8							
		Old	5.37	5.5	3–7							
*PAImpactRatio (%)*	EDL	Young	-0.03	0.09	-0.36–0.19	2.13	0.12	0.13	0.17	0.44	*	Increasing
		Middle-aged	0.34	-0.23	-0.58–0.66							
		Old	0.86	0.33	-0.38–1.28							
	Quadriceps	Young	-0.47	-0.81	-1.34–0.98	7.28	**	0.18	*	2.42	***	Increasing
		Middle-aged	0.5	-0.51	-1.48–0.74							
		Old	4.45	1.18	-0.86–8.35							

### Lunges

**Fig. 4:**
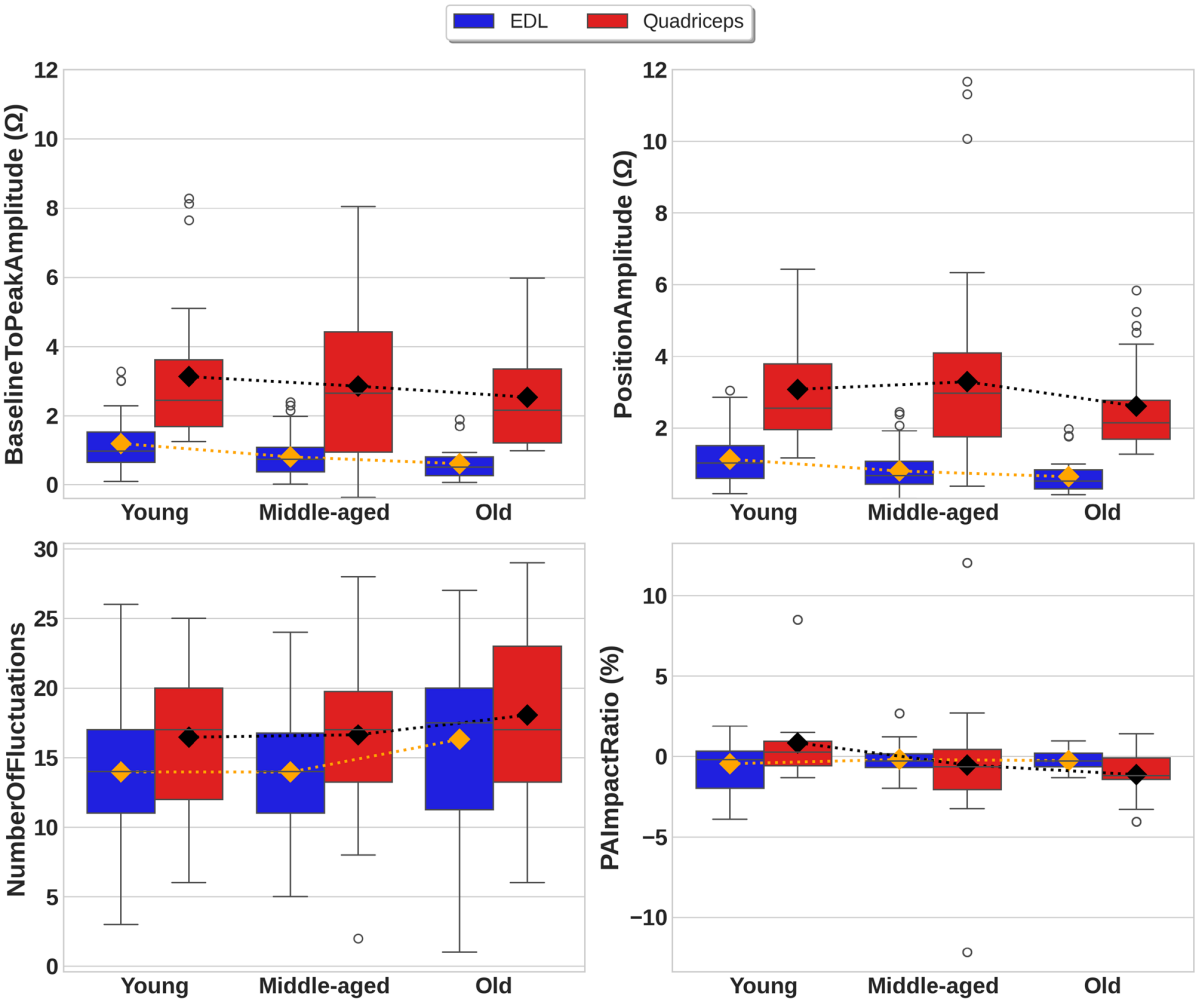
Box plots illustrating the feature distribution across age groups and muscles in the EBI signals obtained during lunges. The diamonds indicate the means and the mean trend lines are indicated by dots.

Figure 4 illustrates boxplots and mean trend dot lines for the *BaselineToPeakAmplitude, PositionAmplitude, NumberOfFluctuations*, and *PAImpactRatio* across different age groups and muscles during lunges.

Both Quadriceps and EDL muscles demonstrated a monotonic decline in mean *BaselineToPeakAmplitude* when aging , consistently exhibiting higher values for the Quadriceps muscle. In contrast, PositionAmplitude displayed a muscle-specific aging pattern; while the EDL mirrored the *BaselineToPeakAmplitude* trend with a monotonic decrease, the Quadriceps muscle showed a non-monotonic change, characterized by an initial increase from young to middle-aged groups, followed by a subsequent decrease in older group. The mean values for *NumberOfFluctuations* showed a non-monotonic trend for the EDL muscle, with a small increase between the mean value for the young group and the middle-aged group, and a larger increase between the mean value for the middle-aged group and the old group. In contrast, the mean values for *NumberOfFluctuations* displayed a steady increase with age for the Quadriceps muscle. The *PAImpactRatio* demonstrated contrasting age-related trends between the Quadriceps and EDL muscles. Specifically, the Quadriceps muscle exhibited a progressive decline in mean *PAImpactRatio* with age, accompanied by a distributional shift from positive to negative values as visualized in boxplots. Conversely, the EDL maintained a relatively stable, consistently negative *PAImpactRatio* distribution across all age groups, showing minimal variation.

Table 6 presents the statistical results obtained for the features across different age groups during lunges.

**Table 6:** Summary of statistical results for features from EBI signals during lunges (* < 0.05, ** < 0.01, *** < 0.001).

Feature	Muscle	Age group	Mean	Median	IQR	ANOVA_F	ANOVA_$\rho$	r	r_$\rho$	$\beta$	$\beta$_$\rho$	Mean trend
*BaselineToPeakAmplitude *($\Omega$)	EDL	Young	1.18	0.97	0.64–1.52	6.57	**	-0.28	**	-0.29	***	Decreasing
		Middle-aged	0.80	0.73	0.37–1.07							
		Old	0.60	0.50	0.26–0.79							
	Quadriceps	Young	3.12	2.44	1.68–3.60	0.74	0.48	-0.11	0.22	-0.30	0.23	Decreasing
		Middle-aged	2.84	2.65	0.94–4.42							
		Old	2.53	2.15	1.20–3.34							
*PositionAmplitude *($\Omega$)	EDL	Young	1.12	1.02	0.58–1.50	5.21	**	-0.28	**	-0.25	**	Decreasing
		Middle-aged	0.79	0.67	0.42–1.06							
		Old	0.63	0.50	0.29–0.82							
	Quadriceps	Young	3.07	2.50	1.95–3.77	1.22	0.30	-0.12	0.21	-0.22	0.35	Non-monotonic
		Middle-aged	3.28	2.96	1.75–4.10							
		Old	2.61	2.14	1.68–2.76							
*NumberOfFluctuations*	EDL	Young	13.97	14	11.00–17.00	2.05	0.13	0.14	0.13	1.14	0.10	Non-monotonic
		Middle-aged	13.97	14	11.00–16.75							
		Old	16.30	17.5	11.25–20.00							
	Quadriceps	Young	16.03	17	12.00–20.00	0.88	0.42	0.09	0.34	0.79	0.24	Increasing
		Middle-aged	16.63	17	13.25–19.75							
		Old	18.07	17	13.25–23.00							
*PAImpactRatio* (%)	EDL	Young	-0.45	-0.19	-1.96–0.34	0.56	0.58	-0.02	0.84	0.10	0.49	Non-monotonic
		Middle-aged	-0.19	-0.28	-0.69–0.16							
		Old	-0.26	-0.27	-0.64–0.21							
	Quadriceps	Young	0.84	0.25	-0.59–0.93	2.89	0.06	-0.33	***	-0.99	*	Decreasing
		Middle-aged	-0.53	-0.64	-2.06–0.44							
		Old	-1.13	-1.19	-1.42–0.09							

The mean values for *BaselineToPeakAmplitude* showed a significant age-related decline for the EDL muscle, with the mean value decreasing from 1.18 $\Omega$ for the young group to 0.6 $\Omega$ for the old group. This decreasing trend was supported by the statistical tests: ANOVA (F = 6.57, $\rho$ = .002); Spearman correlation (r = $-.28$, $\rho$ = .002); and OLS regression ($\beta$ = $-.29$, $\rho$ $<.001$). Also, for the Quadriceps muscle, a decreasing trend was shown, with mean values decreasing from 3.12 $\Omega$ for the young group to 2.53 $\Omega$ for the old group. However, no statistically significant trend was found (ANOVA_F = 0.74, $\rho$ = .48; Spearman_r = $-.11$, $\rho$ = .22; OLS_$\beta$ = $-.30$, $\rho$ = .23). It is also noticeable , for features extracted from the Quadriceps muscle during lunges, the box plots exhibit a consistently wider spread across all age groups compared to the EDL muscle. For instance, the IQR for *BaselineToPeakAmplitude* in the young group was $1.68-3.60$ $\Omega$ for the Quadriceps muscle, whereas it was narrower for the EDL muscle at $0.64-1.52$ $\Omega$. This pattern of greater variability for the Quadriceps muscle is observed across other features as well. In addition to that, despite the visual trend, the low correlation values indicate a weak relationship between age and *BaselineToPeakAmplitude* feature extracted from the Quadriceps muscle.

The mean values for *PositionAmplitude* showed a significant age-related decline for the EDL muscle, with the mean value decreasing from 1.12 $\Omega$ for the young group to 0.63 $\Omega$ for the old group. This trend is supported by the statistical results: ANOVA F = 5.21, $\rho$ = .007; Spearman correlation r = $-.28$, $\rho$ = .002; and OLS regression $\beta$ = $-.25$, $\rho$ = .002. For the Quadriceps muscle, the mean values for *PositionAmplitude* followed a non-monotonic pattern, with the highest mean observed for the middle-aged group (3.28 $\Omega$). However, Statistical results showed no significant trend (ANOVA_F = 1.22, $\rho$ = .30; Spearman_r = $-.12$, $\rho$ = .21; OLS_$\beta$ = $-.22$, $\rho$ = .35). Again, the box plots show greater variability in EBI signals from the Quadriceps muscle, with wider spreads and more outliers across all age groups.

The mean values for *NumberOfFluctuations* displayed a non-monotonic trend for the EDL muscle, with similar mean values observed for the young and middle-aged groups (13.97 fluctuations). Also, there were no statistically significant differences across age groups (ANOVA_F = 2.05, $\rho$ = .13; Spearman_r = .14, $\rho$ = .13; OLS $\beta$ = 1.14, $\rho$ = .10). For the Quadriceps muscle, an increasing trend was observed in the mean values for the *NumberOfFluctuations*, with the mean value rising from 16.03 fluctuations for the young group to 18.07 fluctuations for the old group. However, this trend was not statistically significant (ANOVA_F = 0.88, $\rho$ = .42; Spearman_r = .09, $\rho$ = .34; OLS_$\beta$ = 0.79, $\rho$ = .24). Additionally, the box plots demonstrated that both the EDL and Quadriceps muscles exhibited a consistently wide data spread, with the EBI signal from the Quadriceps muscle showing greater *NumberOfFluctuations* variability compared to the EDL.

The mean values for *PAImpactRatio* exhibited a non-monotonic trend for the EDL muscle, with the highest mean value observed for the middle-aged group ($-0.19$%). No statistically significant trend was found (ANOVA_F = 0.56, $\rho$ = .58; Spearman_r = $-.02$, $\rho$ = .84; OLS $\beta$ = 0.10, $\rho$ = .49). In contrast, the mean values for PA Impact Ratio for the Quadriceps muscle demonstrated a decreasing trend, with a decrease from 0.84% for the young group $-1.13$% to the old group. This trend was supported by a significant Spearman correlation r = $-.33$, $\rho$ $<.001$ and OLS regression $\beta$ = $-0.99$, $\rho$ = .02, although ANOVA did not show a significant difference (F = 2.89, $\rho$ = .06). While the correlation is statistically significant, the r value remains modest, indicating a weak-to-moderate relationship.

### Statistical tests correction

A total of 54 statistical tests were performed across features, muscles, and PAs. After applying the Benjamini-Hochberg procedure, 20 results remained statistically significant, primarily for the EDL muscle across all PAs. Table 7 summarizes the significant findings.

**Table 7:** Summary of statistical correction results using the Benjamini-Hochberg procedure for features from EBI signals across all PAs.

Physical activity	Muscle	Feature	$\rho$-value	Adjusted $\rho$-value
Relaxed standing	EDL	*PrePAmagnitude *($\Omega$)	$<0.001$	$<0.001$
	EDL	*r *(*PrePAmagnitude *($\Omega$))	$<0.001$	$<0.001$
	EDL	*OLS *(*PrePAmagnitude* ($\Omega$))	$<0.001$	$<0.001$
	Quadriceps	*PrePAmagnitude *($\Omega$)	0.01	0.03
Squats	EDL	*BaselineToPeakAmplitude * ($\Omega$)	$<0.001$	$<0.001$
	EDL	*PositionAmplitude *($\Omega$)	0.02	0.04
	EDL	*r *(*PositionAmplitude *($\Omega$))	0.01	0.04
	EDL	*OLS *(*PositionAmplitude *($\Omega$))	$<0.001$	0.01
	EDL	*r* (*BaselineToPeakAmplitude *($\Omega$))	$<0.001$	$<0.001$
	EDL	*OLS *(*BaselineToPeakAmplitude* ($\Omega$))	$<0.001$	$<0.001$
	Quadriceps	*NumberOfFluctuations*	0.02	0.04
	Quadriceps	*PAImpactRatio (%)*	$<0.001$	0.01
	Quadriceps	*OLS (PAImpactRatio (%))*	$<0.001$	$<0.001$
Lunges	EDL	*BaselineToPeakAmplitude *($\Omega$)	$<0.001$	0.01
	EDL	*PositionAmplitude *($\Omega$)	0.01	0.02
	EDL	*r *(*BaselineToPeakAmplitude * ($\Omega$))	$<0.001$	0.01
	EDL	*r* (*PositionAmplitude *($\Omega$))	$<0.001$	0.01
	EDL	*OLS *(*BaselineToPeakAmplitude *($\Omega$))	$<0.001$	$<0.001$
	EDL	*OLS *(*PositionAmplitude* ($\Omega$))	$<0.001$	0.01
	Quadriceps	*r (PAImpactRatio (%))*	$<0.001$	$<0.001$

## Discussion

In our previous study^15^, we showed that the four different PAs exhibited distinct EBI signal characteristics. Also, the results showed that variations in characteristics of each PAs occur due to the participant execute the PAs in respect to speed, step length, depth, and corrections for balance. In this study, we investigated the capability of EBI technology to evaluate age-related differences in muscle condition and function during distinct PAs (i.e., relaxed standing position, squats, and lunges) in a cohort of 39 participants.

Some of the old and middle-aged participants had an interest in being informed about their fall risk. This led to a partial recruitment bias toward participants primarily concerned with their mobility and stability. The study itself focused on muscle function where mobility and stability are key components. As a reference, also young participants were recruited. This provided a targeted population well-suited for exploring age-related muscle changes. This focus offered a justified and appropriate foundation for assessing the potential of EBI to differentiate between PAs patterns across age groups. Moreover, the initial sample enabled a preliminary evaluation of EBI’s capabilities and supported the refinement of experimental procedures, establishing a basis for future studies with larger and more diverse cohorts.

The relaxed standing position measurement served as a reference condition, providing a baseline evaluation of muscular properties prior to dynamic activity. The statistical analysis revealed significant age-related differences in EBI features for both the EDL and Quadriceps muscles. For the EDL muscle, a consistent monotonic decline with 17% in the *PrePAmagnitude* was observed between the young group and the old group. This pattern reflects the progressive loss of muscle mass, strength, and function associated with Sarcopenia^17,18^. This decline is primarily driven by the loss of Type II (fast-twitch) muscle fibers, which are predominant in the EDL muscle^18–20^.The age-related reduction in EBI signal therefore highlights a loss of muscle tissue volume and supports the notion that aging impairs muscle excitability and function^17,21^. Interestingly, the Quadriceps muscle exhibited a non-monotonic trend across age groups, with elevated impedance values observed in the middle-aged group compared to both the young and old groups. Despite this nonlinear pattern, a 21% decrease in the *PrePAmagnitude* from the young group to the old group was confirmed by multiple statistical tests, indicating a significant age-related decline. However, the observed elevation in the middle-aged group was modest (mean: 26.31 $\Omega$) compared to the young group (mean: 25.64 $\Omega$), and both values fall within overlapping IQRs ($19.31-32.61$ and $21.85-32.61$, respectively). This suggests that the difference may reflect natural inter-individual variability or transitional physiological adaptations rather than a distinct shift in muscle composition. This activity-dependent variation may suggests a compensatory mechanism, potentially involving increased recruitment of Type I (slow-twitch) muscle fibers, which are more resistant to age-related deterioration, and are predominant in postural and endurance muscles such as the Quadriceps muscle^18,20^.

The dynamic activity revealing the most pronounced age-related differences was squats. Out of the four features per muscle (i.e., a total of eight features), there was a statistically significant age difference (ANOVA) for five out of eight features during squats and two out of eight features during lunges. The differing statistical outcomes between squats and lunges likely stem from variations in biomechanical complexity and muscle activation patterns. Although lunges are more demanding due to their asymmetrical nature and higher balance requirements, squats showed more consistent and significant age-related differences in EBI features. This may be due to their standardized execution, symmetrical loading, and more uniform muscle engagement-especially for the EDL and Quadriceps muscles-making age-related muscular changes easier to detect. Magnitude-based features such as *BaselineToPeakAmplitude* and *PositionAmplitude*, reflecting muscle mass and contraction dynamics respectively, exhibited monotonic declines with age in the EDL muscle across both dynamic PAs, i.e., squats and lunges. The mean trend reductions were slightly more pronounced during lunges, with *BaselineToPeakAmplitude* decreasing by 49% between the young group and the old group, and *PositionAmplitude* by 44% between the young group and the old group. For squats, *BaselineToPeakAmplitude* decreased by 47% and *PositionAmplitude* by 36% between the young group and the old group. This indicates that the EDL muscle function deteriorates consistently with age, regardless of the PA’s complexity. In contrast, the Quadriceps muscle showed more variable trends, with *PositionAmplitude* being the only significantly differing feature during squats (7% decrease between the young group and old group), and no statistically significant differences were observed during lunges. This divergence may be attributed to the Quadriceps muscle’s role as a postural and endurance muscle, rich in Type I muscle fibers, which are more resistant to age-related decline.

Age-dependent differences in muscle fatigue were evident through the *PAImpactRatio*, which measures post-activity impedance changes. During squats, the old group showed a significant increase in the *PAImpactRatio* of the Quadriceps muscle, reaching a 1047% rise compared to the young group, indicating increased post-exertional impedance and greater muscle fatigue accumulation. Conversely, during lunges, a substantial, though statistically non-significant via ANOVA, an age-related decrease of 235% in the *PAImpactRatio* was observed between the young group and old group. These variations likely reflect age-related alterations in muscle hydration^22,23^, extracellular matrix remodeling^24,25^, and recovery capacity^26,27^, with the higher muscular demands of squats exacerbating these effects. Additionally, structural changes such as fibrosis^28^, fat infiltration^29,30^, and altered water content^31,32^, may contribute to the observed impedance differences in the old group.

The results for the dynamic PAs mirrored the trends observed during the relaxed standing position, particularly for the EDL muscle. Across both squats and lunges, magnitude-based features demonstrated consistent and monotonic declines with age, reinforcing the pattern seen at relaxed standing position measurement. This reflects a consistent decrease in the amplitude of the EDL muscle with age, likely due to a shift to Type II muscle fibers. In contrast, the Quadriceps muscle did not exhibit such monotonic behavior. Its age-related trends were more inconsistent and PA-dependent, both at rest and during dynamic tasks. The non-monotonic pattern suggests gradual, time-dependent changes in muscle composition. These changes may reflect transitional physiological adaptations, such as compensatory fiber recruitment or muscle remodeling, potentially involving a progressive loss of Type II muscle fibers and a relative shift toward Type I muscle fibers in muscles^20^. However, due to the cross-sectional nature of the study and lack of direct fiber-type composition data, the exact timing and mechanisms of this shift remain unclear. Greater age-group granularity would be necessary to track how the Quadriceps muscle changes with age and identify when these non-monotonic transitions happen. The *NumberOfFluctuations* feature, reflecting neuromuscular adjustments for balance and coordination, showed clear age- and PAs-dependent patterns. As expected, the inherently more complex lunge required more frequent adjustments (more fluctuations) to keep the balance than the squat across all groups, highlighting the feature’s sensitivity to balance demands. The EDL muscle demonstrated stable *NumberOfFluctuations* across age groups in both dynamic PAs, likely because its supportive role is less impacted by age-related coordination changes during these specific movements. Conversely, the Quadriceps muscle showed a significant age-related effect during squats, where the non-monotonic pattern suggests that the individuals in the middle-aged group might employ more frequent compensatory adjustments to counteract early declines in their balance and coordination, while old individuals may adopt different, possibly less dynamic or adaptive, control strategies. The complexity of lunges likely masked clear age-related effects in both muscles, unlike the more controlled squat where patterns were easier to see. Therefore, fluctuation analysis reveals that aging affects neuromuscular coordination in complex, non-monotonic ways that are specific to the muscle’s function and the PA’s stability requirements.

The application of the Benjamini-Hochberg procedure allowed us to identify statistically significant results while controlling for false discoveries across multiple comparisons. Notably, most significant findings were observed for the EDL muscle, particularly for magnitude-based features such as *PrePAmagnitude* and *BaselineToPeakAmplitude*. These results reinforce the sensitivity of EBI features to age-related muscular changes. The Quadriceps muscle showed fewer significant results, consistent with its more non-monotonic behavior across age groups. The use of FDR correction supports the robustness of our findings while maintaining exploratory sensitivity

Although we did not evaluate different sensor locations, our findings clearly show that sensor location significantly influences the interpretation of results. Sensors located on smaller, less-trained muscles like the EDL muscle, provide clearer insights into intrinsic age-related decline, while those placed on commonly trained muscles, such as the Quadriceps muscle, primarily reflect a person’s training level and the current shape and condition of those muscles. The result obtained reveal that the young group displayed greater muscle function and adaptability, characterized by healthy tissue structure, efficient contraction, and minimal muscle post-PA fatigue. In the middle-aged group, however, muscle responses differed: the Quadriceps muscle showed signs of compensatory activity, whereas the EDL muscle presented early functional decline, indicating a shift towards a transitional neuromuscular state. The old group demonstrated uniform deterioration across all EBI features, consistent with advancing muscle mass loss and reduced neuromuscular control. Together, these findings underscore the importance of muscle selection for sensor location in evaluating age-related changes in muscle function and adaptation.

Indeed, the age-related decline in muscle function observed in the EBI signals aligns with well-established physiological changes. The results suggest that measuring EBI signals could serve as a tool to assess early markers for deteriorated mobility that occurs when a person ages. Detection of changes could allow for interventions that mitigate the progression of disability and improve quality of life. This proactive measurement approach could enhance clinical practices by providing quantifiable data on muscle health among the middle-aged adults and further on when individuals age^13,33–35^. However, while these interpretations are consistent with known physiological mechanisms, it is important to acknowledge that EBI signals represent integrated tissue responses rather than isolated measures of muscle mass^36–38^. The current study does not include direct measurements of muscle composition, fiber-type distribution, or motor unit characteristics. Therefore, our EBI findings should be interpreted as reflecting the combined effects of multiple age-related neuromuscular and tissue changes rather than singular pathophysiological processes^12,35,39^. Therefore, the proposed explanations, such as fiber-type shifts or compensatory neuromuscular strategies, should be viewed as plausible hypotheses rather than definitive conclusions. Future studies incorporating longitudinal data could investigate the validity of this hypothesis.

Several important limitations of the study should be noted. The results are based on 39 participants who were divided into three age groups for analytical purposes. Convenience sampling resulted in uneven distribution across for example age, gender, physical fitness, cultural, physiological in-heritage, and body composition. Therefore, the results are not generalizable to the whole population. Future controlled studies with balanced demographics and comprehensive body assessment will help isolate pure aging effects from demographic influences. Additionally, several other factors may have impacted on the results of this study: (i) Placement of the electrode on selected muscles; (ii) Execution of exercises in a controlled environment; (iii) Repeating the PAs on different occasions prior to data collection might have yielded other results in terms of for example speed, power, and balance.

Another limitation of our study is that only one type of sensor is used. To better understand individual differences during PAs, data from other types of sensors and equipment could be collected and analyzed. While this study focuses exclusively on EBI-based evaluation, we fully acknowledge the importance of comparing EBI with established tools such as EMG, muscle strength testing and body composition analysis. Access to such information might help contextualizing EBI findings and validate their relevance in evaluating muscle age differences.

## Conclusions

This study highlights the potential of EBI for assessing age-related muscle function decline. The analysis of EBI features obtained from 39 participants (aged 20–73) during relaxed standing position, squats and lunges reveals a progressive reduction in muscle constitution and neuromuscular coordination which is consistent with known aging effects. For squats, the majority of features were significantly different across age groups, which make it a promising PA for detecting neuromuscular aging effects. For lunges, only 25% of the features were significantly different across age groups, likely due to its higher biomechanical complexity. The EDL muscle showed a consistent age-related decline across all three PAs, while changes for the Quadriceps muscle were more inconsistent and PA-dependent. EBI emerges as a promising non-invasive tool for monitoring neuromuscular aging, with potential applications in early muscle mass loss detection and rehabilitation monitoring.

## Data Availability

The data supporting the conclusions of this article will be made available by the corresponding author, S.Z., upon request, without undue reservation.
